# Solid state fermentation process with *Aspergillus kawachii* enhances the cancer-suppressive potential of silkworm larva in hepatocellular carcinoma cells

**DOI:** 10.1186/s12906-019-2649-7

**Published:** 2019-09-05

**Authors:** Hyun-Dong Cho, Hye-Ji Min, Yeong-Seon Won, Hee-Young Ahn, Young-Su Cho, Kwon-Il Seo

**Affiliations:** 1 Industry-Academy Cooperation, Dong-A University, 49315, Busan, Republic of Korea; 2 Department of Biotechnology, Dong-A University, 37, Nakdong-daero 550 Street, Saha-gu, Busan, 49315 Republic of Korea; 3 Institute of Agriculture Life Science, 49315 Busan, Republic of Korea

**Keywords:** Apoptosis, Cell cycle arrest, Fermentation, Silkworm larvae, Human hepatocellular carcinoma

## Abstract

**Background:**

Mulberry silkworm larvae (*Bombyx mori*) are known as the oldest resource of food and traditional medicine. Although silkworm larvae have been reported to treat various chronic diseases, the effect of fermentation by microorganisms improving the biological activities of silkworm larvae was not reported. In the present study, fermented silkworm larvae was developed via solid-state fermentation with *Aspergillus kawachii* and investigated its anti-cancer activity in human hepatocellular carcinoma cells.

**Methods:**

We investigated the anti-cancer effects of unfermented (SEE) and fermented silkworm larva ethanol extract (FSEE) on HepG2 human hepatocellular carcinoma cells as well as compared changes in free amino acid, fatty acid, and mineral contents. Anti-cancer activities were evaluated by SRB staining, cell cycle analysis, Annexin V staining, Hoechst staining, DNA fragmentation analysis and western blot analysis. Fatty acid, free amino acid and mineral contents of SEE and FSEE were determined by gas chromatography, amino acid analyzer and flame atomic absorption spectrophotometer, respectively.

**Results:**

Compared with SEE, treatment with FSEE resulted in apoptotic cell death in HepG2 cells characterized by G0/G1 phase cell cycle arrest, DNA fragmentation, and formation of apoptotic bodies. Furthermore, FSEE significantly up-regulated pro-apoptotic as well as down-regulated anti-apoptotic proteins in HepG2 cells. However, an equivalent concentration of SEE did not induce cell cycle arrest or apoptosis in HepG2 cells. Moreover, fermentation process by *Aspergillus kawachii* resulted in enhancement of fatty acid contents in silkworm larvae, whereas amino acid and mineral contents were decreased.

**Conclusion:**

Collectively, this study demonstrates that silkworm larvae solid state-fermented by *Aspergillus kawachii* strongly potentiates caspase-dependent and -independent apoptosis pathways in human hepatocellular carcinoma cells by regulating secondary metabolites.

**Electronic supplementary material:**

The online version of this article (10.1186/s12906-019-2649-7) contains supplementary material, which is available to authorized users.

## Background

Microorganisms have long been implicated in the production of fermented foods (fish, dairy, meat, legumes, and various vegetables) and alcoholic beverages. Edible microorganisms, including enzymes, amylases, proteases, and lipases, hydrolyze macromolecules in food ingredients into small saccharides, amino acids, and fatty acids with specific flavors, aromas, and textures [1]. *Aspergillus kawachii*, classified as a member of the black *Aspergilli* fungi group, has been used in Korea and Japan as an enzymatic source for traditional *shochu-koji* and wine from rice or barley grain [2]. During fermentation, diverse hydrolytic enzymes, including amylase, protease, and lipase, are activated by *Aspergillus kawachii* in metabolomics (*kawachii* lipase) [3, 4]. In recent years, there has been increasing interest in the development of fermented products derived from microorganisms, as they are more safe, acceptable, nutritious, and effective in terms of health than processed ones containing chemical compounds. In addition, these fermented products are very useful for various applications in the medicinal perspective, such as natural product medicine, and development of functional foods.

The continuously increasing world population has incentivized the development of novel food sources with high feed conversion efficiency, low space requirements, and nutritive excellence. In some parts of the world, consumption of insects is traditionally practiced since they are highly nutritious and good sources of proteins, fat, minerals, and vitamins [5]. Mulberry silkworm (*Bombyx mori*) is known as the oldest domesticated insect in the world for producing silk, and it is consumed as a food and traditional medicine [6]. Silkworm larvae have been confirmed by recent studies as consisting of 25–30% fat, 55–60% proteins, 2–4% saccharides, and trace elements and vitamins [7]. Silkworm larvae have also been reported to have various beneficial activities such as anti-cancer [8], anti-inflammatory [9], antioxidant [10], and anti-obesity [11]. Further, numerous studies have reported that bioconversion or fermentation performed by microorganisms can be used to improve the biological activities of natural products by enhancing secondary metabolite composition [12, 13]. Thus, bioconverted or fermented edible insects are more likely to be effective for treating various chronic diseases than unprocessed ones.

According to the American Cancer Society®, liver cancer is the fifth leading cause of cancer death in men and the eighth leading cause of cancer death in women. It is estimated that liver cancer accounted for about 41,000 new cases and 29,000 cancer deaths in the United States in 2017. Numerous inducing factors, including chronic viral hepatitis (Hep-B or Hep-c), aflatoxins, smoking, fatty liver, cirrhosis, alcohol, obesity, and type 2 diabetes, are related with liver cancer, and the incidence of liver cancer has gradually increased worldwide. After liver cancer is diagnosed, chemotherapy with drugs is typically used to destroy cancer cells [14]. However, distributed chemotherapeutic agents in the body can affect normal cell toxicities as well as induce significant side effects, including hair loss, mouth sores, loss of appetite, nausea, vomiting, and diarrhea [15]. Therefore, numerous studies have attempted to identify low-toxicity anti-cancer therapeutics derived from natural products as well as to understand their molecular mechanisms for cancer suppression.

Tumorigenesis can be induced by uncontrolled cell proliferation coordinating a number of protein mechanisms. Cell cycle machinery, which controls the genomic progression of cells, involves a subset of CDK-cyclin complexes consisting of three interphase CDKs (CDK2, CDK4 and CDK6), a mitotic CDK (CDK1) and 10 cyclins belonging to four different classes (−A, −B, −D, and –E type cyclins). Exogenous and endogenous genotoxic agents activate DNA damage checkpoints to allow repair of DNA defects as well as up-regulate CDK inhibitors, including p21, p27, and p57, which induce cell cycle arrest through modulation of CDK activity [16]. Nowadays, there is evidence regarding the role of apoptotic cell death, which is directly linked to genes involved in cancer cell cycle arrest such as p53 [17]. Apoptosis, known as programmed cell death, can also be defined as a cell death mechanism that controls unwanted cell development. Generally, apoptosis is initiated via extrinsic and intrinsic pathways, resulting in various morphological and biochemical changes such as DNA fragmentation, chromatin condensation, and formation of apoptotic bodies [18]. Since failure to induce cancer cell death is known to be an important cause of malignant tumors [19], cell cycle arrest and apoptosis induction could be useful targets for cancer treatment. However, the apoptotic mechanism induced by fermented silkworm larvae in various cancer cells remains unclear, and there has been no comparative study on the cancer suppressive effects of fermented and unfermented silkworm larvae.

Therefore, the present study investigated the anti-cancer activities of unfermented and fermented silkworm larvae in human liver cancer cells as well as elucidated underlying molecular mechanisms. In addition, the results demonstrate for the first time that silkworm larvae fermented by *Aspergillus kawachii* showed significant changes in fatty acid, free amino acid, and mineral contents compared with unfermented silkworm larvae. Collectively, we aimed to obtain direct evidence of the remarkable cancer inhibitory activity of fermented silkworm larvae ethanol extract (FSEE) in human liver cancer cells as compared to unfermented silkworm larvae ethanol extract (SEE) as well as to determine possible mechanisms of action.

## Methods

### Chemicals

The general caspase inhibitor (z-vad-fmk), and apoptosis-inducing factor (AIF) inhibitor (N-phenylmaleimide, N-PM) was obtained from R&D systems (Minneapolis, MN, USA), and Sigma-Aldrich Co. Ltd. (st. Louis, USA). Anti-Bax (sc-7480), anti-Bcl-2 (sc-7382), anti-caspase-3 (sc-7272), anti-caspase-8 (sc-7890), anti-caspase-9 (sc-7885), anti-AIF (sc-5586), anti-poly (ADPribose) polymerase-1 (PARP-1) (sc-7150), anti-p53 (sc-47698), anti-p21 (sc-24559), anti-cyclin-dependent kinase (CDK) 2 (sc-6248), anti-CDK4 (sc70831), anti-cyclin D1 (sc-70899), and anti-*β*-actin (sc-47778) antibodies were purchased from Santa Cruz Biotechnology (Santa Cruz, CA, USA). Mitochondria isolation kit and bicinchoninic acid (BCA) protein assay kit were purchased from Pierce (Rockford, IL, USA). ECL kit was ordered from Amersham Life Science (Amersham, UK).

### Preparation of fermented and unfermented silkworm larvae extract

The dried silkworm larvae used in this study were supplied from local market (Busan, Republic of Korea). Silkworm larvae species authentication was performed by Dr. Kwang-Sik Lee, professor of college of natural resources and life science (industrial entomology), Dong-A University. *Aspergillus kawachii* KCCM 32819 were purchased from Korean Culture Center of Microorganism (KCCM). Seed culture of *Aspergillus kawachii* was prepared by inoculating a loopful of spores from a potato dextrose (PD) broth slant into 500 mL of PD medium (pH 5.0), and shake-cultured at 30 °C for 72 h with 150 rpm.

Preparation of fermented silkworm larvae were performed five times by solid state fermentation in that order. Dried silkworm larvae were cut and crushed in a mechanical juicer, and sterilized at 121 °C for 15 min. After then 5% (v/w) of seed-cultured *Aspergillus kawachii* without culture medium was inoculated to 20 g of sterilized silkworm larvae powder, and cultured in an incubator at 30 °C for 0 day (unfermented silkworm larvae) and 3 days (fermented silkworm larvae). Silkworm larvae unfermented, and fermented by *Aspergillus kawachii* was dried at 60 °C to obtain dried sample.

The unfermented, and fermented silkworm larvae were extracted as described. The 10 g of dried unfermented, and fermented silkworm larvae was extracted three times with 100 mL of distilled water, and ethanol at 37 °C for 12 h. After the extracts had centrifuged (3000 rpm, 5 min), the clear supernatant was filtered with a 0.45 μm pore size polytetrafluoroethylene filter (Merck KGaA, Darmstadt, Germany), and concentrated by vacuum evaporation. The yield of extract was 206.16 mg/g (unfermented silkworm larvae water extract), 217.61 mg/g (fermented silkworm larvae water extract), 401.28 mg/g (unfermented silkworm larvae ethanol extract), and 415.23 mg/g (fermented silkworm larvae ethanol extract). Unfermented, and fermented silkworm larvae water and ethanol extracts were dissolved in DMSO, and then diluted with cell culture medium to make required concentration for identifying in vitro anti-cancer activity.

### Fatty acid contents determination

The total fatty acids in unfermented, and fermented silkworm larvae were extracted according to the Folch, Lees, & Stanley [20] method with some modifications. Briefly, 1 g of fermented silkworm larvae powder were extracted at 37 °C for 30 min using chloroform: methanol 2:1 (v/v) containing butylated hydroxyl toluene to inhibit the oxidation of fatty acids. Following centrifugation (3000×*g*, 15 min), the part of chloroform was concentrated in rotary vacuum evaporator to remove chloroform solvent. Extracted fatty acids were trans methylated to the fatty acid methyl esters by adding 5 mL of methanolic HCl (5:1, v/v) at 65 °C for 3 h. The fatty acid methyl esters were analyzed using gas chromatography (GC-17A, Shimadzu Co., Kyoto, Japan), equipped with an omegawax® capillary GC column (30 m × 0.25 mm × d_f_ 0.25 μm). Helium was the carrier gas, and the column flow rate was 1 mL/min. One μL of sample was injected in splitless mode into an inlet held at 250 °C, and the oven program was 180 °C. The data are expressed as mg/g of detecting total analyzed fatty acids.

### Free amino acid contents determination

Free amino acids were extracted from unfermented, and fermented silkworm larvae powder according to the previous reports [21]. Fermented silkworm larvae powder (1 g) was mixed with 20 mL of hexane for removing fat at 30 °C in an ultrasonic homogenizer for 24 h. The defatted samples were blended with 10 mL of ethanol (70%) and shaken with a rotary shaker for 1 h. After the solution had centrifuged (10,000×*g*, 15 min), supernatant was filtered through a 0.2 μm pore size membrane filter (Whatman Inc., Dassel, Germany). Then the solvents were removed using vacuum evaporator, and reconstituted with 0.2 M sodium citrate buffer (pH 2.2) to a final volume (5 mL). The free amino acid was analyzed by Biochrom 30 series amino acid analyzer (Biochrom Ltd., Cambridge, UK) according to the protocol. The free amino acid was post-column derivatized with ninhydrin reagent and detected by absorbance at 570 nm.

### Mineral contents determination

The mineral contents of unfermented, and fermented silkworm larvae were determined using a method of AOAC [22]. Briefly, 1 g of unfermented and fermented silkworm larvae powder was ashed in porcelain crucibles at 500 °C for 12 h. Dry ashed samples were dissolved in 10 mL of deionized water, and sequentially filtered using 0.2 μm membrane filter (Whatman Inc., Dassel, Germany). Sodium (Na), zinc (Zn), magnesium (Mg), manganese (Mn), calcium (Ca), iron (Fe), and potassium (K) contents in the sample were measured using an AAnalyst 300 flame atomic absorption spectrophotometer (Perkin Elmer, Waltham, MA, USA). Minerals were determined using the instrumental conditions recommended for each mineral and were calculated based on the respective standard curve. Data values were expressed as μg/g sample weight.

### Cell culture and proliferation

The HepG2 human hepatocellular carcinoma cells were purchased from American Type Culture Collection (ATCC, Manassas, VA, USA). The cells were cultured in Dulbecco modified eagle medium (DMEM) supplemented with 10% fetal bovine serum (FBS), penicillin (100 IU/mL), and streptomycin (100 μg/mL) (Gibco, ThermoFisher Scientific Co., Waltham, MA, USA). The cells were incubated in an incubator containing a humidified atmosphere of 5% CO_2_ at 37 °C.

### Sulforhodamine B (SRB) assay

Cell proliferation was determined by sulforhodamin B (SRB, Sigma, St. Louis, USA) assay. The HepG2 cells were seeded at a concentration of 3 × 10^4^ cells/well in 48-well tissue culture plates and incubated with various concentrations of SE and FSE for 24 h. After treatment, medium was aspirated and 10% trichloro-acetic acid was added. After 1 h incubation at 4 °C, the plate was washed five times with D. W and air-dried. The cells were stained with 0.4% (w/v) SRB at room temperature for 1 h and then washed five times using 1% acetic acid. Bound SRB was solubilized with 10 mM Tris, and the absorbance was measured at 540 nm using a microplate reader (Molecular Devices Inc., Sunnyvale, CA, USA).

The influence of z-vad-fmk (caspases inhibitor), and N-PM (N- phenylmaleimide, AIF inhibitor) on cell viability was also determined by SRB assay. The cells were seeded at a densities of 3 × 10^4^ cells per well in a 24-well plate, and then cultured for 24 h in DMEM. The cells were pre-incubated with 10 μM of z-vad-fmk or 2 μM of N-PM for 2 h and then treated with the indicated concentrations of SEE, and FSEE for 24 h. SRB assay was conducted as described above.

### Cell cycle analysis

The cell cycle phases were determined using Muse^Ⓡ^ cell cycle kit reagent, according to the manufacturer’s protocol. 1 × 10^6^ HepG2 cells were seeded into 6 well plates and incubated for 24 h. Treatments were given with various concentrations of SE, and FSE for 24 h. The cells were trypsinized and washed with PBS. One mL of 70% ethanol added to microcentrifuge tubes and then incubated for 3 h at − 20 °C. Finally, 200 μL of the reagent added to each tube and incubated for 30 min at room temperature. The cells were analyzed using a Muse cell analyzer (Merck KGaA, Darmstadt, Germany). The flow cytometry data was obtained from 5000 events (gated cells) per sample. The percentages of cells shown in the figures were calculated from the mean fluorescence intensity in each of the four quadrants. In addition, the coefficient of variation from the mean fluorescence was less than 10%.

### Annexin V staining assay

The apoptotic cell death was determined using Muse^Ⓡ^ Annexin V and dead cell reagent, according to the manufacturer’s protocol. 1 × 10^5^ prostate tumor and prostate epithelial cells were seeded into 24 well plates and incubated for 24 h. Treatments were given with various concentrations of extracts of SEE, and FSEE for 24 h. The cells were trypsinized and washed with PBS. Added 100 μL of the reagent to microcentrifuge tubes and then added 10 μL of cell suspension to each tube and incubated for 20 min at room temperature. The cells were analyzed using a Muse cell analyzer (Merck KGaA, Darmstadt, Germany). The flow cytometry data was obtained from 5000 events (gated cells) per sample. The percentages of cells shown in the figures were calculated from the mean fluorescence intensity in each of the four quadrants. In addition, the coefficient of variation from the mean fluorescence was less than 10%.

### Detection of morphological apoptosis

Characteristic apoptotic morphological changes were assessed by fluorescent microscopy using bis-benzimide (Hoechst 33258) staining. Briefly, the cells were seeded in 6-well plates at a density of 1 × 10^6^ cells per well, followed by treatment with SEE and FSEE for 24 h. After harvesting, the cells were washed twice with PBS and then stained with 200 μL of bis-benzimide (5 μg/mL) for 10 min at room temperature. Then, 10 μL of this suspension was placed on a glass slide and covered with a cover slip. The cells were examined using a fluorescence microscope (Olympus Optical Co. Ltd. Japan) to determine nuclei fragmentation and chromatin condensation.

### Analysis of DNA fragmentation

The HepG2 cells were seeded at a density of 2 × 10^6^ cells in a 100 mm dish and cultured for 24 h in DMEM. After culturing, the cells were treated with the indicated concentrations of SEE and FSEE for 24 h, followed by centrifugation. The pellets were lysed by lysis buffer (10 mM Tris–HCl, pH 7.5, 10 mM EDTA, pH 8.0, 0.5% Triton X-100, 20% SDS, and 10 mg/mL of proteinase K) and then centrifuged. After extraction with phenol: chloroform: isoamyl alcohol (25:24:1), DNA was precipitated with 2 vol of cold absolute ethanol. The resulting pellets were incubated with TE buffer (10 mM Tris–HCl, pH 7.4, 1 mM EDTA, pH 8.0) and RNase (2 mg/mL) for 1 h at 37 °C. Then, separation by electrophoresis was performed on 2% agarose containing ethidium bromide. The resulting DNA bands were examined using a UV Trans illuminator Imaging System.

### Mitochondria isolation

Mitochondria fraction was isolated from cell lysate using a Mitochondria isolation kit (Pierce, Rockford, USA). The HepG2 cells were seeded at a density of 5 × 10^6^ cells in a 100 mm dish, and then cultured for 24 h in DMEM. After culturing, the cells were treated with the indicated concentrations of SEE and FSEE for 24 h, followed by centrifugation. Next, 800 μL of Reagent A and 10 μL of Reagent B were added to 2 × 10^6^ cell pellet and incubated on ice for 2 min. The resulting pellets were centrifuged at 700×*g* for 10 min at 4 °C. The supernatant was then transferred to a new tube and centrifuged at 12000×*g* for 15 min at 4 °C. The supernatant (cytosol fraction) was added to a new tube, and the pellet containing mitochondria received 500 μL of Reagent C followed by centrifugation (12,000×*g*, 5 min, 4 °C). The mitochondria pellets were lysed by lysis buffer (50 mM Tris-HCl, 150 mM NaCl, 1 mM EDTA, 50 mM NaF, 30 mM Na_4_P_2_O_7_, 1 mM PMSF, and 2 μg/mL of aprotinin) for 30 min on ice. Protein expression in the mitochondria fraction was analyzed by western blot assay.

### Western blot analysis

The HepG2 cells were seeded at a density of 5 × 10^6^ cells in a 100 mm dish, and then cultured for 24 h in DMEM. After culturing, the cells were treated with the indicated concentrations of SEE and FSEE for 24 h, followed by centrifugation. The resulting pellets were lysed by lysis buffer (50 mM Tris-HCl, 150 mM NaCl, 1 mM EDTA, 50 mM NaF, 30 mM Na_4_P_2_O_7_, 1 mM PMSF, and 2 μg/mL of aprotinin) for 30 min on ice. The protein content of the supernatant was measured using a BCA protein kit (Pierce, Rockford, IL, USA). The protein samples were then loaded at 10 μg of protein/lane and then separated by 12% SDS-PAGE at 100 V of constant voltage/slab for 1.5 h. Following electrophoresis, the proteins were transferred onto nitrocellulose membranes. After blocking with 2.5 and 5% BSA for 1 h at 37 °C, the membranes were incubated with primary antibody at 4 °C overnight. Finally, the membranes were treated with horseradish peroxidase-coupled secondary antibodies for 1 h at 4 °C. The membranes were then washed with T-TBS after each antibody binding reaction. Detection of each protein was performed using an ECL kit (Santa Cruz, CA, USA). The intensity of each band was quantified by Image studio™ Lite software (LI-COR Inc., NE, USA), and the fold of increase was presented comparing with β-actin.

### Statistical analysis

The statistical analyses were evaluated by one-way analysis of variance (ANOVA), with differences analyzed using the Duncan’s new multiple-range test. Levels of *p < 0.05, **p < 0.01, and ***p < 0.001 were regarded as statistically significant.

## Results

### Effects of SEE and FSEE on proliferation of human hepatocellular carcinoma cells

**Fig. 1:**
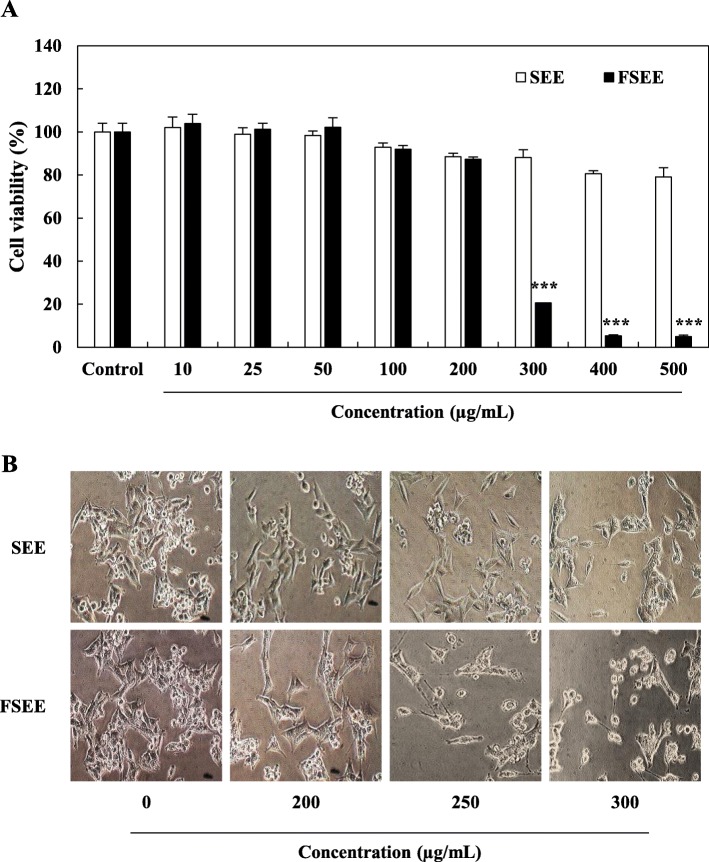
Cell growth inhibitory effects on HepG2 hepatocellular carcinoma cells treated with silkworm larva ethanol extract (SEE), and fermented silkworm larva ethanol extract (FSEE) for 24 h. **a** Cell viability was measured by SRB assay. Data values were expressed as mean ± SD of triplicate determinations. Significant differences were compared with the control at *p < 0.05, **p < 0.01, and ***p < 0.001 using one-way ANOVA. **b** After 24 h incubation with SEE, and FSEE, cell morphology was visualized by inverted microscopy (× 200)

In our previous study, we tested anticancer activity improvement of silkworm larvae extract by fermentation process using various microorganisms. Among the six microorganisms, fermented silkworm larvae by *A. kawachii* (FSEE) showed highest anticancer activity in human hepatocellular carcinoma cells (Additional file 1: Figure S1). In order to investigate the inhibitory effects of treatment with SEE and FSEE on proliferation of human liver cancer cells, HepG2 cells were treated with various doses of SEE and FSEE (Fig. 1). The effects of SEE and FSEE on suppression of HepG2 cell viability were confirmed by SRB assay. As shown in Fig. 1a, HepG2 cells treated with 10–500 μg/mL of SEE did not showed remarkable changes in cell proliferation. However, HepG2 cells treated with 300–500 μg/mL of FSEE showed a significant decrease in cell growth at 24 h compared with control group cells. In FSEE-treated HepG2 cells, morphological changes such as cell shrinkage, rounding, and detachment were observed, whereas non-significant effects were observed in SEE-treated HepG2 cells (Fig. 1b). Meanwhile, fermented silkworm larvae water extract (FSWE) did not show any significant cytotoxicity in HepG2 cells when compared with FSEE treatment (Additional file 2: Figure S2). These results demonstrate that FSEE but not SEE treatment suppressed the viability and proliferation of HepG2 human hepatocellular carcinoma cells.

### Effects of SEE and FSEE on cell cycle arrest in human hepatocellular carcinoma cells

**Fig. 2:**
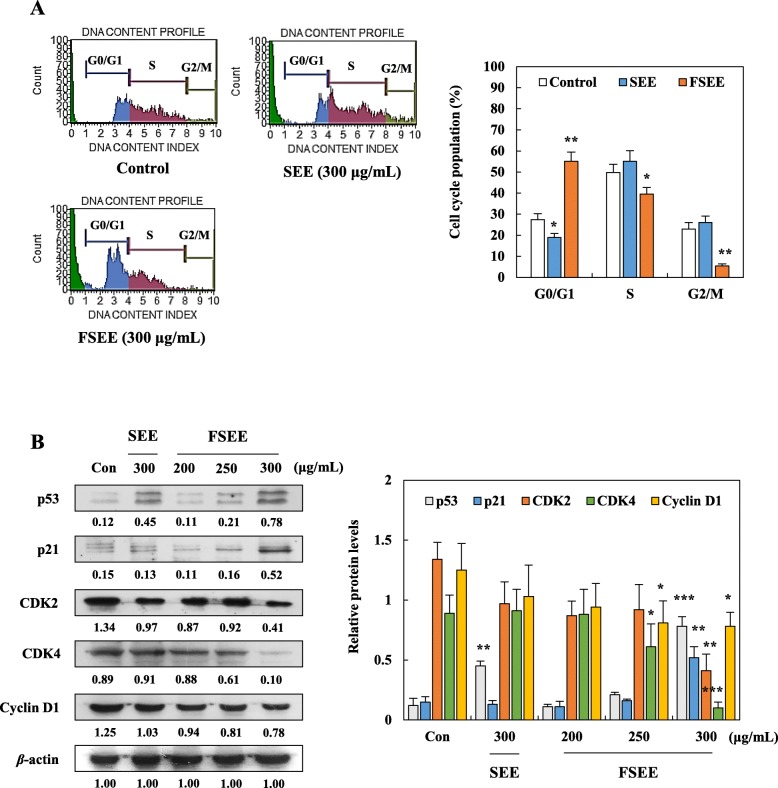
Effect of SEE, and FSEE on G0/G1 cell cycle arrest in HepG2 hepatocellular carcinoma cells. Cells were treated with SEE, and FSEE for 24 h. **a** Cell cycle population was analyzed by flow cytometry. **b** After 24 h, total cell lysates were subjected to detect expression of cell cycle arrest-related proteins in HepG2 cells. Data values were expressed as mean ± SD of triplicate determinations. Significant differences were compared with control at *p < 0.05, **p < 0.01, and ***p < 0.001 using one-way ANOVA

Since unscheduled cancer cell proliferation is caused by continuous re-entry into cell cycle progress [16], we conducted cell cycle analysis in SEE- and FSEE-treated HepG2 cells. As shown in Fig. 2a, G0/G1, S, and G2/M phases of the cell cycle showed a normal distribution in both control and 300 μg/mL of SEE-treated HepG2 cells. However, following exposure to 300 μg/mL of FSEE for 24 h, G0/G1 phase of HepG2 cells was up-regulated by 28.39% compared to control cells, whereas S and G2/M phases of HepG2 cells were down-regulated by 10.48 and 18.19%, respectively. Furthermore, FSEE-induced accumulation of G0/G1 phase cells was shown to be related with regulation of the expression of proteins playing important roles in cell cycle progression (Fig. 2b). Treatment with FSEE resulted in down-regulation of cyclin D1, CDK 2, and CDK 4 as well as up-regulation of p21 and p53 in a dose-dependent manner (Fig. 2b). In SEE-treated HepG2 cells were slightly decreased G0/G1 population in HepG2 cell cycle and enhanced expression of p53, but not changed p21, CDK2, CDK4, cyclin D1. These findings reveal that FSEE suppressed proliferation of HepG2 cells by inducing G0/G1 phase arrest.

### Effects of SEE and FSEE on induction of apoptosis in human hepatocellular carcinoma cells

**Fig. 3:**
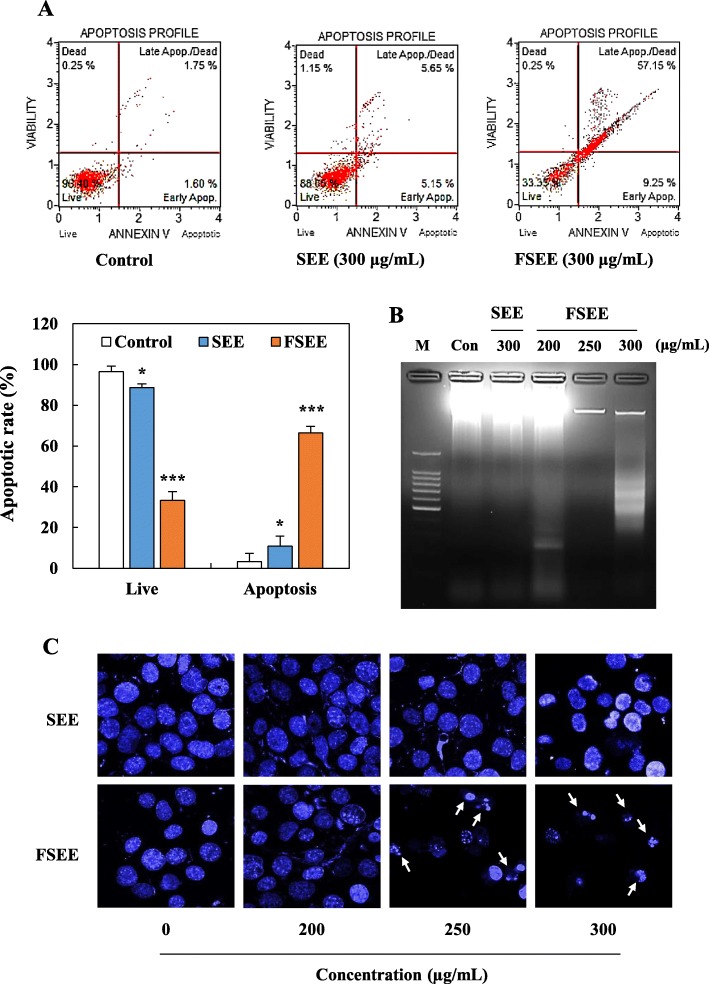
Effect of SEE, and FSEE on induction of apoptotic cell death in HepG2 hepatocellular carcinoma. Cells were treated with SEE, and FSEE for 24 h. **a** Early and late apoptosis population was analyzed by Annexin V staining assay. **b** DNA fragmentation was observed by 2% agarose gel electrophoresis. **c** Nuclear condensation in response to SEE, and FSEE treatment as detected by Hoechst staining assay. Data values were expressed as mean ± SD of triplicate determinations. Significant differences were compared with control at *p < 0.05, **p < 0.01, and ***p < 0.001 using one-way ANOVA

In order to determine whether or not the anti-proliferative effect of FSEE is associated with apoptotic cell death, we further performed Annexin V analysis, DNA fragmentation, and Hoechst staining assay. As demonstrated in Fig. 3, treatment with SEE had no significant effect on the apoptotic cell population (Fig. 3a), DNA fragmentation (Fig. 3b), or morphological changes (Fig. 3c) in HepG2 cells. However, HepG2 cells incubated with 300 μg/mL of FSEE for 24 h showed a remarkably increased apoptotic cell population (Fig. 3a), DNA fragmentation (Fig. 3b), and nucleic condensation (Fig. 3c) when compared to non-treated and SEE-treated cells. These data show that FSEE induced apoptotic cell death in HepG2 human hepatocellular carcinoma cells.

### Effects of SEE and FSEE on induction of apoptosis via a mitochondrial apoptosis pathway in human hepatocellular carcinoma cells

**Fig. 4:**
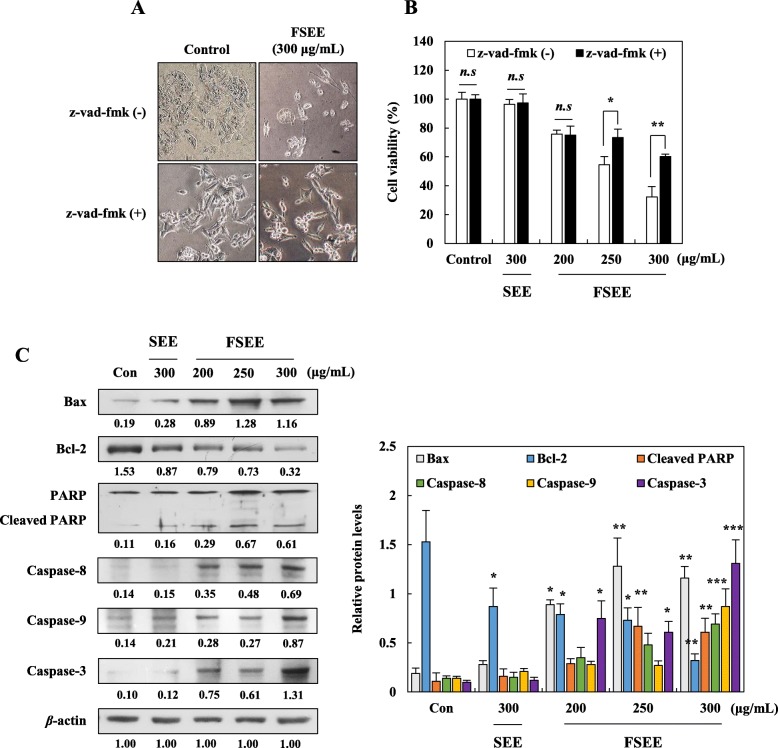
Effect of SEE, and FSEE on caspase-dependent apoptosis by activating the mitochondrial apoptotic pathways in HepG2 cells. Cells were pretreated with 10 μM z-vad-fmk for 2 h, and then incubated with 300 μg/mL of SEE, and 200–300 μg/mL of FSEE for 24 h. Effects of the caspase inhibitor on (**a**) SEE, and FSEE-induced morphological changes, and (**b**) cell death in HepG2 cells were examined. After 24 h treatment, (**c**) total cell lysates were subjected to detect expression of apoptosis-related proteins in HepG2 cells. Data values were expressed as mean ± SD of triplicate determinations. Significant differences were compared with control at *p < 0.05, **p < 0.01, and ***p < 0.001 using one-way ANOVA

Activation of caspases is believed to be a receptor-regulated effect induced in response to cellular stress or an increase in cytosolic calcium [18]. To determine whether or not FSEE-induced apoptosis is stimulated by a caspase-dependent pathway, the effects of SEE and FSEE on caspase-mediated apoptotic cell death were examined by SRB assay using the broad caspase inhibitor z-vad-fmk (Fig. 4a, b). Pretreatment of cells with z-vad-fmk significantly blocked FSEE-induced cell death without any morphological changes, and co-treatment with SEE and z-vad-fmk did not show cytotoxicity in HepG2 cells (Fig. 4a, b). Furthermore, the effects of SEE and FSEE on expression of mitochondria-mediated apoptotic pathway proteins were investigated by Western blot analysis. As shown in Fig. 4c, treatment with FSEE increased expression of pro-apoptotic Bax and caspases-8, − 9, and − 3 proteins as well as suppressed expression of anti-apoptotic Bcl-2 proteins. Moreover, up-regulation of cleaved-PARP was observed in HepG2 cells after treatment with FSEE. However, an equivalent concentration of SEE did not induce significant changes in the expression of proteins compared with control cells. These results imply that FSEE-induced apoptosis was regulated by a mitochondrial apoptosis signaling pathway.

### Effects of SEE and FSEE on release of cytochrome c and AIF from the mitochondria into the cytosol in human hepatocellular carcinoma cells

**Fig. 5:**
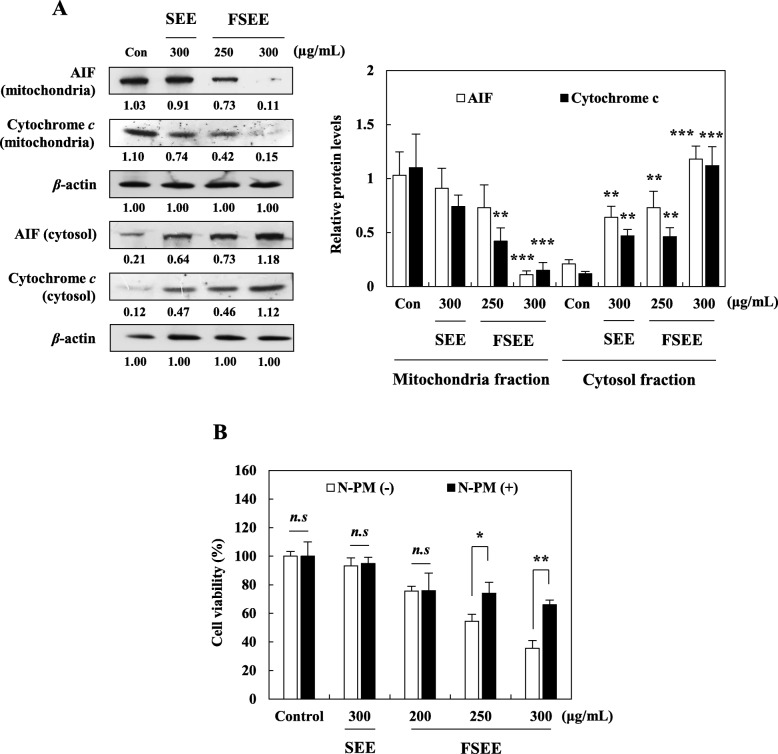
Effect of SEE, and FSEE on induction of caspase-independent apoptosis in HepG2 cells. Cells were treated with SEE, and FSEE for 24 h. **a** After 24 h, total cell lysates were subjected to detect expression of apoptosis-related proteins in HepG2 cells. Cells were pretreated with 2 μM AIF inhibitor (N-PM) for 2 h, and then incubated with 300 μg/mL of SEE, and 200–300 μg/mL of FSEE for 24 h. **b** Effect of the AIF inhibitor on SEE, and FSEE-induced cell death in HepG2 cells was examined by SRB assay. Data values were expressed as mean ± SD of triplicate determinations. Significant differences were compared with control at *p < 0.05, **p < 0.01, and ***p < 0.001 using one-way ANOVA

It is well known that release of cytochrome c and AIF from mitochondria into the cytosol plays important events in caspase-dependent and -independent apoptosis pathways [23, 24]. To further validate whether or not FSEE activates caspase-independent proteins for induction of apoptosis, expression levels of AIF and cytochrome c were measured by Western blot analysis. The results show that release of AIF and cytochrome c from mitochondria into the cytosol was elevated in FSEE-treated HepG2 cells in a dose-dependent manner, whereas treatment with SEE at a concentration of 300 μg/mL resulted in similar expression levels of AIF and cytochrome c compared to control cells (Fig. 5a). Moreover, pretreatment with AIF inhibitor (N-PM) gradually suppressed FSEE-induced apoptotic cell death in HepG2 cells (Fig. 5b). Treatment or co-treatment of HepG2 cells with SEE and N-PM did not affect cell viability (Fig. 5b). These data support the idea that FSEE induced apoptotic cell death in HepG2 cells through both caspase-dependent and -independent pathways.

### Chemical changes in fermented and unfermented silkworm larvae in terms of free amino acid, fatty acid, and mineral contents

To compare chemical composition between unfermented and fermented silkworm larvae fermented by *Aspergillus kawachii*, fatty acid, free amino acid, and mineral contents were analyzed. Fatty acid contents of fermented and unfermented silkworm larvae are given in Table 1. In unfermented silkworm larvae, linolenic acid was the most abundant with a concentration of 103.65 mg/g, followed by oleic acid (85.22 mg/g), palmitic acid (48.81 mg/g), stearic acid (26.34 mg/g), and linoleic acid (21.79 mg/g). Total fatty acid contents of fermented and unfermented silkworm larvae were 285.81 and 356.85 mg/g, respectively. Among them, contents of palmitic acid, palmitoleic acid, stearic acid, oleic acid, linoleic acid, and arachidonic acid in silkworm larvae were enhanced by fermentation using *Aspergillus kawachii*, whereas linolenic acid was down-regulated in fermented silkworm larvae. Caprylic acid, decanoic acid, and myristic acid were not detected in fermented or unfermented silkworm larvae.

**Table 1:** Fatty acid content of unfermented, and fermented silkworm. (Unit: mg/g)

Fatty acid	Concentration (mg/g)	
	Unfermented silkworm	Fermented silkworm
Caprylic acid (8:0)	ND	ND
Decanoic acid (10:0)	ND	ND
Myristic acid (14:0)	ND	ND
Palmitic acid (16:0)	48.81	67.58
Palmitoleic acid (16:1)	ND	0.56
Stearic acid (18:0)	26.34	35.75
Oleic acid (18:1)	85.22	107.69
Linoleic acid (18:2)	21.79	50.39
Linolenic acid (18:3)	103.65	94.88
Arachidonic acid (20:0)	ND	0.25
Total fatty acid	285.81	356.85

The free amino acid compositions of fermented and unfermented silkworm larvae are shown in Table 2. Regarding the amino acid composition of unfermented silkworm larvae, glutamic acid was the most predominant at a concentration of 3.17 mg/g, followed by asparagine (3.06 mg/g), arginine (2.66 mg/g), histidine (2.17 mg/g), alanine (1.45 mg/g), and glycine (1.31 mg/g), whereas remaining amino acids were observed in minor amounts (< 1 mg/g). Compared to unfermented silkworm larvae, contents of taurine, L-aspartic acid, L-threonine, L-valine, L-methionine, L-isoleucine, L-leucine, L-tyrosine, L-phenylalanine, L-cystine, L-proline, L-alanine, L-citrulline, and L-orinithine were elevated in fermented silkworm larvae. However, total free amino acid amounts of fermented silkworm larvae were lower compared to unfermented ones by 3.78 mg/g (Table 2).

**Table 2:** Free amino acid contents of unfermented, and fermented silkworm

Free amino acid	Concentration (mg/g)	
	Unfermented silkworm	Fermented silkworm
Phosphoserine	0.17	0.08
Taurine	–	0.12
Urea	–	–
L-Aspartic acid	0.20	0.23
L-Threonine	0.49	0.76
L-Serine	1.08	0.49
L-Glutamic acid	3.17	3.05
L-Valine	0.43	1.31
L-Methionine	–	0.01
L-Isoleucine	0.24	1.08
L-Leucine	0.37	1.42
L-Tyrosine	0.71	0.92
L-Phenylalanine	1.17	1.39
γ-amino-n-butyric acid	0.02	–
Phosphoethanolamine	0.50	–
L-lysine	0.38	0.04
1-Methyl-L-Histidine	–	0.29
L-Histidine	2.17	0.22
L-Arginine	2.66	0.07
L-Cystine	–	0.03
Asparagine	3.06	–
L-α-Aminoadipic acid	0.22	0.05
L-Proline	0.28	0.38
L-Glycine	1.31	0.42
L-Alanine	1.45	1.75
L-Citrulline	–	0.13
L-α-Aminobutyric acid	0.02	–
L-Orinithine	0.18	2.26
Total free amino acid	20.28	16.50

The major minerals in fermented and unfermented silkworm larvae were Na, Zn, Mg, Mn, Ca, Fe, and K (Table 3). In comparison to unfermented silkworm larvae, silkworm larvae fermented with *Aspergillus kawachii* resulted in down-regulation of various minerals, including Na, Zn, Mg, Mn, Ca, Fe, and K, by 14.64–94.28%. Together, these results demonstrate that the fermentation process using *Aspergillus kawachii* enhanced fatty acid contents in silkworm larvae but reduced free amino acid and mineral contents.

**Table 3:** Mineral content of unfermented, and fermented silkworm. (Unit: μg/g)

Mineral	Concentration (μg/g)	
	Unfermented silkworm	Fermented silkworm
Na	0.70	0.04
Zn	0.76	0.64
Mg	58.00	49.67
Mn	0.41	0.35
Ca	68.00	1.85
Fe	0.82	0.60
K	316.30	76.30
Total mineral content	445.00	129.47

## Discussion

In this study, FSEE prepared by solid state fermentation using *Aspergillus kawachii* inhibited multiple apoptosis signaling pathways in HepG2 human hepatocellular carcinoma cells. Proliferation of HepG2 cells incubated with FSEE was suppressed via cell cycle arrest at G0/G1 phase, and apoptosis was induced in a caspase-dependent and -independent manner. In addition, the involvement of chemical composition changes in free amino acid, fatty acid, and mineral contents during fermentation in FSEE-induced apoptotic cell death was demonstrated for the first time. Therefore, the results of the present study provide novel evidence of FSEE as a natural anti-cancer material, suggesting its potential as a natural medicine for the attenuation of liver cancer development.

In recent years, increasing global demand for food substitutes has brought attention to insects due to their abundance of nutrients and potential as a valuable new food source [25]. Since silkworm larvae is the most thoroughly domesticated insect for commercial silk production, it is not surprising that silkworm larvae have been widely used as a food and animal feed worldwide. Numerous studies have indicated that silkworm larvae (*Bombyx mori* and *Samia ricinii*) containing high quality crude protein (50–70%), fat (8–26%), crude fiber (3–6%), and crude ash (4–11%) is a rich source of nutrients [5, 26]. In addition, the fatty acid composition of silkworm pupae has been reported as follows: 0.1–0.2% myristic acid, 23.2–24.2% palmitic acid, 1.7% palmitoleic acid, 4.5–4.7% stearic acid, 26.0–28.3% oleic acid, 3.9–7.3% linoleic acid, and 36.3–38.3% linolenic acid. The amino acid content of silkworm larvae has been reported as follows: 10.7% Asp, 4.6% Thr, 4.3% Ser, 11.1% Glu, 4.2% Gly, 4.6% Ala, 1.7% Cys, 5.6% Val, 4.0% Met, 4.0% Ile, 7.3% Leu, 6.6% Tyr, 5.4% Phe, 7.2% Lys, 3.2% His, 5.5% Arg, 8.2% Pro, and 1.8% Trp [25, 27]. In the present study, the amino acid and fatty acid compositions of unfermented silkworm larvae were similar to those of previous reports. The most predominant mineral type of unfermented silkworm larvae was found to be K with a concentration of 316.30 μg/g, representing approximately 71% of total mineral contents, followed by Ca (68.00 μg/g) and Mg (58.00 μg/g). However, the mineral contents of unfermented silkworm larvae were lower than those of previous reports that investigated mineral amounts in four varieties of silkworm larvae [28]. Differences in amino acid, fatty acid, and mineral contents between silkworm larvae are believed to be due to harvest period, species, feed type, and growth environment.

Effective processing techniques have received great interest in the food and medicine industry due to their ability to maintain biological activities and numerous benefits. Although several studies have reported the nutrient compositions of silkworm larvae and pupae [7, 25, 27, 28], little information is available on compositional changes induced by fermentation or bioconversion. In our results, fermentation process a slight decrease in the free amino acid and mineral contents of silkworm larvae. Sudo et al. [29] reported that *Aspergillus kawachii* needs carbon, nitrogen, minerals, and oxygen for mycelial growth, suggesting that amino acids and minerals in unfermented silkworm larvae are consumed during fermentation. Interestingly, palmitic acid, stearic acid, oleic acid, and linoleic acid were shown to be up-regulated by 40.79, 37.50, 26.69, and 139.95% during fermentation. Filamentous fungi have been traditionally used as a food resource in the forms of miso, cheese, and alcoholic beverages due to the high efficiency of various kinds of enzyme production [30]. Especially, well-known fungi such as *Penicillium restrictum*, *Canadida rugose*, *Colletotrichum gloeosporioides*, *Aspergillus niger*, and *Sporobolomyces ruberrimus* have been reported to produce fungal lipases, which catalyze hydrolysis of triglycerides to fatty acids and glycerol [31]. In addition, lipase production of filamentous fungi in solid state fermentation is affected by numerous factors such as culture media, nitrogen sources, oils, carbohydrates, pH, O_2_, CO_2_, temperature, and metal ions [31, 32]. These results indicate that *Aspergillus kawachii* hydrolyzed the rich lipid proportion of silkworm larvae to increase fatty acid contents during the fermentation process. However, reduction of free amino acid and mineral contents by fermentation suggests that *Aspergillus kawachii* utilized the amino acids and minerals for metabolism in fermentation.

Cell cycle progression and arrest in eukaryotic cells are regulated by balance among CDKs, CDK inhibitors, and cyclins [33]. Regarding the cell cycle machinery, the mammalian cell cycle controls specific CDK-cyclin complexes for driving sequential and orderly cell cycle events. For instance, the first G1/S phase checkpoint controls two types of kinase complexes, CDK4/6-cyclin D and CDK2-cyclin E, which regulate entry into DNA synthesis S phase [16]. Hyperproliferative diseases such as cancer invariably suffer from defective cell cycle checkpoint function caused by genetic mutations or dysfunctions in important molecular proteins [33]. In these diseases, activation of checkpoints based on DNA damage and cell size can effectively disrupt progression of the cell cycle and cause development of cancer. Based on this view, treatment with FSEE inhibited proliferation of HepG2 human hepatocellular carcinoma cells by promoting cell cycle arrest in G0/G1 phase. Furthermore, expression levels of cyclin D1 and CDK2/4 were down-regulated after treatment with 300 μg/mL of FSEE for 24 h, and CDK inhibitors such as p53 and p21 were up-regulated in HepG2 cells incubated with FSEE. Although SEE induced slight decrease of G0/G1 phase of cell cycle and increase of p53 expression, SEE did not affect induction of apoptosis and cell growth inhibitory effect. These data show that fermentation of silkworm larvae improved cancer inhibitory activity through cell cycle arrest in G0/G1 phase.

Tumor cells maintain their resistance to cell death by overexpression of anti-apoptotic Bcl-2 and suppression of pro-apoptotic Bax, which is regulated by p53 tumor suppressor [34]. However, the stress signals from DNA damage and/or death receptor-ligand systems cause mitochondrial permeability transition, followed by release of cytochrome c, AIF, and endo G from the mitochondrial inter-membrane space into the cytosol [35]. In the cytosol, cytochrome c combines with caspase-9 to assemble a cytoplasmic complex known as the apoptosome, which induces intrinsic apoptosis. Furthermore, up-regulated AIF and endo G relocate to the nucleus and play important roles in facilitating large-scale DNA degradation in a caspase-independent manner. According to our results, FSEE-induced apoptotic cell death was shown to be related with caspase-dependent and -independent pathways. Pretreatment with a broad caspase inhibitor (z-vad-fmk) and AIF inhibitor (N-PM) significantly reduced FSEE-induced apoptotic cell death. Further, remarkable down-regulation of Bcl-2, up-regulation of Bax and caspases-8, − 9, and − 3, as well as cleavage of PARP proteins were observed after treatment with FSEE for 24 h. Cytosolic AIF and cytochrome c were also up-regulated in FSEE-treated HepG2 cells, whereas mitochondrial AIF and cytochrome c were down-regulated in a dose-dependent manner. Since alteration of apoptotic signaling is believed to cause tumor resistance to cancer therapies, recent anti-cancer therapies have focused on inducing tumor cell death through various apoptosis mechanisms [34]. These data indicate that FSEE could induce apoptosis through diverse modes, including caspase-dependent and -independent signaling pathways, suggesting potential treatment strategies for apoptosis-resistant types of cancer.

Several studies have reported that changes in the secondary metabolites and nutrient compositions of natural products induced by fermentation are beneficial for human health based on their biological activities [36, 37]. Although slight increase of total phenolic content was observed in FSEE, the increase rate was only 3.17% compared to SEE (from 3.46 to 3.57 mg GAE/g). However, water extracts of unfermented or fermented silkworm larvae (SWE and FSWE) indicated highest total phenolic contents (4.11 and 4.24 mg GAE/g) among silkworm larvae extracts (Additional file 3: Table S1). Most of phenolic compounds in silkworm larvae might be derived from mulberry leaf [38], however, we can find no dramatic change in phenolic compounds through fermentation process. The current work also shows that fermentation of silkworm larvae using *Aspegillus kawachii* increased fatty acid contents, including palmitic acid, stearic acid, oleic acid, and linoleic acid, compared to unfermented silkworm larvae. Especially, HepG2 human hepatocellular carcinoma cells incubated with FSEE at a concentration of 300 μg/mL for 24 h displayed significantly enhanced caspase-dependent and -independent apoptosis, whereas SEE did not affect cell proliferation. Interestingly, our screening data reveal that the same dose of water extract of fermented silkworm larvae (FSWE) had no significant effect on proliferation of HepG2 cells (Additional file 2: Figure S2). These results indicate that the cancer suppressive activity of FSEE in human hepatocellular carcinoma cells was due to fat-soluble bioactive components, not a phenolic compound. Commonly, fatty acid uptake is considered as an essential step for delivering energy sources to various cells. Besides their well-known roles as energy resources, palmitic acid [39], stearic acid [40], oleic acid [41], linoleic acid [42], and linolenic acid [43] have been shown to have cancer suppressive activities with regards to several cancer cells. Therefore, our findings demonstrate that the marked increase in fatty acid contents in solid state-fermented silkworm larvae by *Aspergillus kawachii* may be attributed to significant apoptotic cell death in HepG2 cells.

## Conclusions

These results demonstrate that fermentation of silkworm larvae using *Aspergillus kawachii* may lead to up-regulation of fatty acids as well as potentiate the anti-cancer activity of silkworm larvae in HepG2 human hepatocellular carcinoma cells. Although further studies are necessary to elucidate bioactive components and tumor suppressive activity, these data expand our understanding that fermentation of silkworm larvae using *Aspergillus kawachii* can enhance apoptotic cell death in human hepatocellular carcinoma cells by regulating secondary metabolites. Therefore, we propose that fermented silkworm larvae using *Aspergillus kawachii* are a novel natural medicine for the prevention and treatment of cancer.

## Additional files

Additional file 1: **Figure S1.** Cell growth inhibitory effects on HepG2 hepatocellular carcinoma cells treated with 300 μg/mL of fermented silkworm larvae water and ethanol extract for 24 h. Cell viability was measured by SRB assay. Data values were expressed as mean ± SD of triplicate determinations. Significant differences were compared with the control at *p < 0.05, **p < 0.01, and ***p < 0.001 using one-way ANOVA. (PPTX 38 kb)

Additional file 2: **Figure S2.** Cell growth inhibitory effects on HepG2 hepatocellular carcinoma cells treated with fermented silkworm larva water extract (FSWE) and fermented silkworm larva ethanol extract (FSEE) for 24 h. Cell viability was measured by SRB assay. Data values were expressed as mean ± SD of triplicate determinations. Significant differences were compared with the control at *p < 0.05, **p < 0.01, and ***p < 0.001 using one-way ANOVA. (PPTX 38 kb)

Additional file 3: **Table S3.** Total polyphenol content from unfermented and fermented silkworm larvae extract. (DOCX 15 kb)

## Data Availability

The supporting materials can be obtained upon request via email to the corresponding author.

## Abbreviations

AIF: Apoptosis-Inducing Factor
ANOVA: Analysis of Variance
ATCC: American Type Culture Collection
BCA: Bicinchoninic Acid
CDK: Cyclin-Dependent Kinase
DMEM: Dulbecco Modified Eagle Medium
FBS: Fetal Bovine Serum
FSEE: Fermented Silkworm larvae Ethanol Extract
FSWE: Fermented Silkworm larvae Water Extract
KCCM: Korean Culture Center of Microorganism
PARP: Poly (ADP ribose) Polymerase
PD: Potato Dextrose
SEE: Silkworm larvae Ethanol Extract
SRB: Sulforhodamin B
