# Supplementary material for: Solid state fermentation process with Aspergillus kawachii enhances the cancer-suppressive potential of silkworm larva in hepatocellular carcinoma cells
Source: BMC Complement Altern Med. 2019 Sep 5;19:241. doi: 10.1186/s12906-019-2649-7 (PMC6727413; doi:10.1186/s12906-019-2649-7)
Supplement: Supplementary file 1 — Figure S1. Cell growth inhibitory effects on HepG2 hepatocellular carcinoma cells treated with 300 μg/mL of fermented silkworm larvae water and ethanol extract for 24 h. Cell viability was measured by SRB assay. Data values were expressed as mean ± SD of triplicate determinations. Significant differences were compared with the control at *p < 0.05, **p < 0.01, and ***p < 0.001 using one-way ANOVA. (PPTX 38 kb) [file 12906_2019_2649_MOESM1_ESM.pptx]

## Slide 1
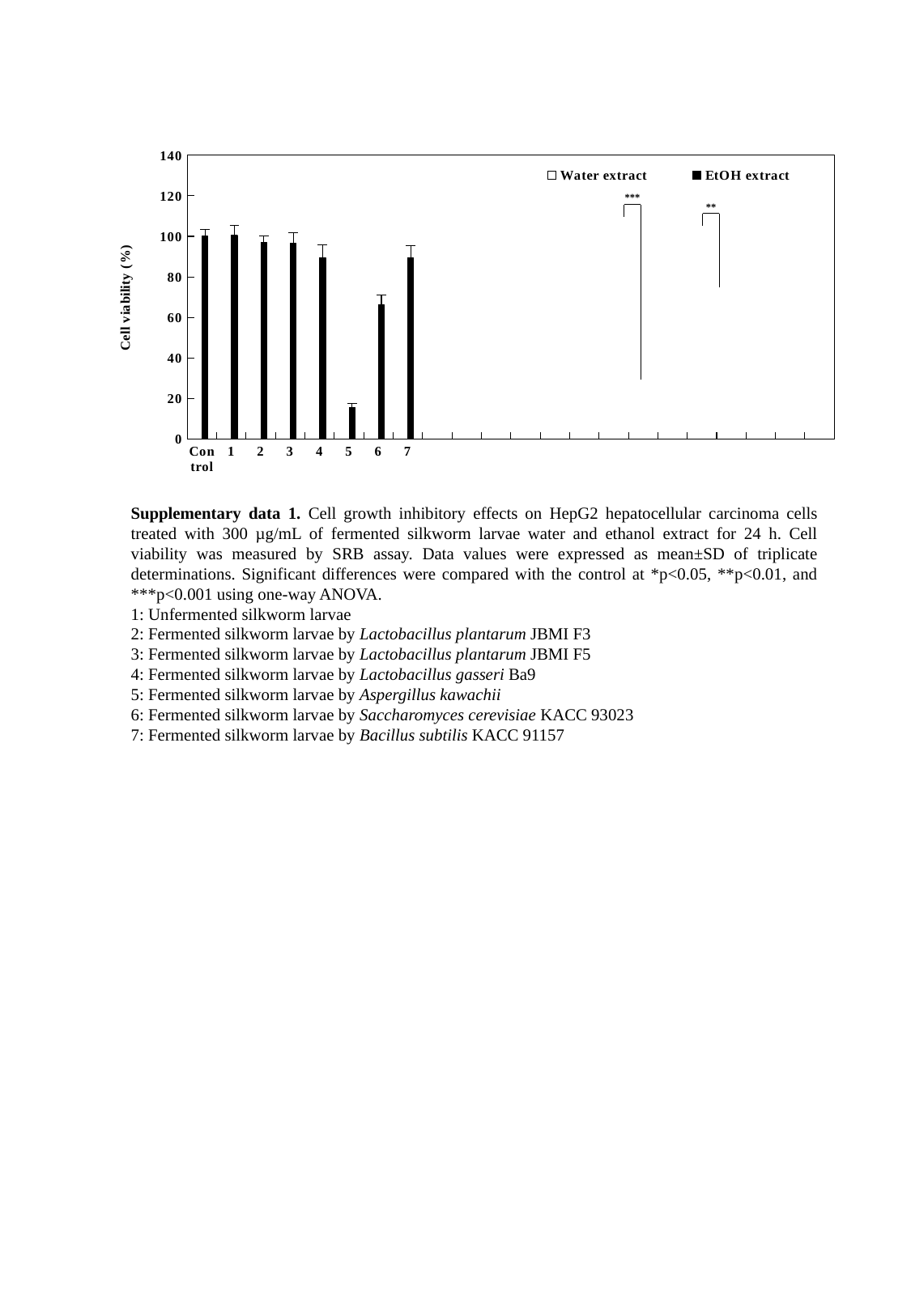

### Chart
| Category | Water extract | EtOH extract |
|---|---|---|
| Control | 100.0 | 100.0 |
| 1 | 92.37 | 100.376 |
| 2 | 101.27 | 97.12 |
| 3 | 94.2376 | 96.45 |
| 4 | 96.2376 | 89.37 |
| 5 | 102.69 | 15.36 |
| 6 | 97.19 | 66.136 |
| 7 | 98.75 | 89.26 |***
**
Supplementary data 1. Cell growth inhibitory effects on HepG2 hepatocellular carcinoma cells treated with 300 µg/mL of fermented silkworm larvae water and ethanol extract for 24 h. Cell viability was measured by SRB assay. Data values were expressed as mean±SD of triplicate determinations. Significant differences were compared with the control at *p<0.05, **p<0.01, and ***p<0.001 using one-way ANOVA.
1: Unfermented silkworm larvae
2: Fermented silkworm larvae by Lactobacillus plantarum JBMI F3
3: Fermented silkworm larvae by Lactobacillus plantarum JBMI F5
4: Fermented silkworm larvae by Lactobacillus gasseri Ba9
5: Fermented silkworm larvae by Aspergillus kawachii
6: Fermented silkworm larvae by Saccharomyces cerevisiae KACC 93023
7: Fermented silkworm larvae by Bacillus subtilis KACC 91157
