# Supplementary material for: Solid state fermentation process with Aspergillus kawachii enhances the cancer-suppressive potential of silkworm larva in hepatocellular carcinoma cells
Source: BMC Complement Altern Med. 2019 Sep 5;19:241. doi: 10.1186/s12906-019-2649-7 (PMC6727413; doi:10.1186/s12906-019-2649-7)
Supplement: Supplementary file 2 — Figure S2. Cell growth inhibitory effects on HepG2 hepatocellular carcinoma cells treated with fermented silkworm larva water extract (FSWE) and fermented silkworm larva ethanol extract (FSEE) for 24 h. Cell viability was measured by SRB assay. Data values were expressed as mean ± SD of triplicate determinations. Significant differences were compared with the control at *p < 0.05, **p < 0.01, and ***p < 0.001 using one-way ANOVA. (PPTX 38 kb) [file 12906_2019_2649_MOESM2_ESM.pptx]

## Slide 1
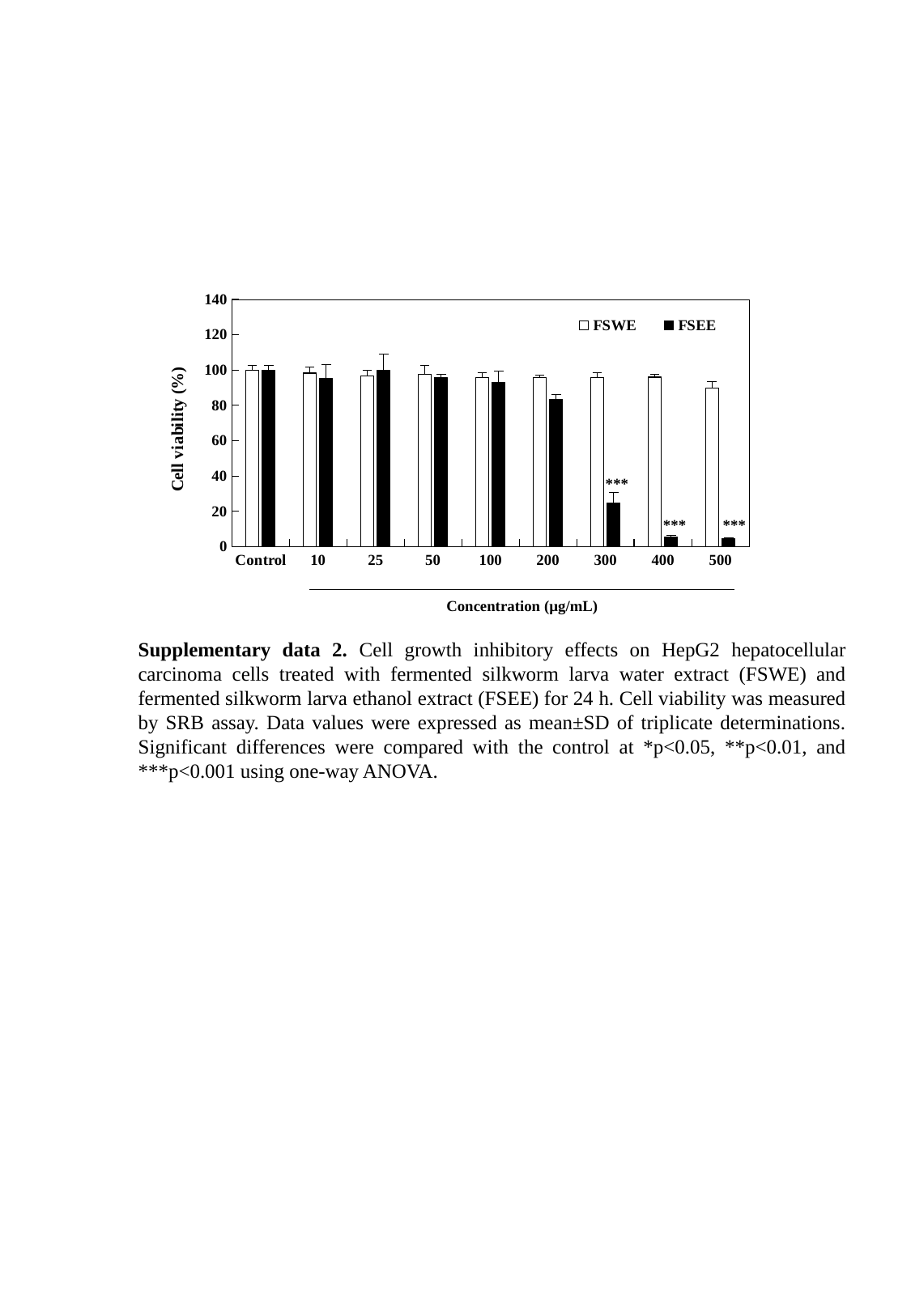

### Chart
| Category | FSWE | FSEE |
|---|---|---|
| Control | 100.0 | 100.0 |
| 10 | 98.29366861248316 | 95.15042658284688 |
| 25 | 96.6995958688819 | 99.88774135608442 |
| 50 | 97.5078581050741 | 95.64436461607545 |
| 100 | 95.5770094297261 | 93.10731926358329 |
| 200 | 95.57700942972609 | 83.56533453075888 |
| 300 | 95.7117198024248 | 24.786708576560397 |
| 400 | 95.98114054782219 | 5.500673551863493 |
| 500 | 89.94162550516391 | 4.467894027840144 |***
***
***
Concentration (µg/mL)
Supplementary data 2. Cell growth inhibitory effects on HepG2 hepatocellular carcinoma cells treated with fermented silkworm larva water extract (FSWE) and fermented silkworm larva ethanol extract (FSEE) for 24 h. Cell viability was measured by SRB assay. Data values were expressed as mean±SD of triplicate determinations. Significant differences were compared with the control at *p<0.05, **p<0.01, and ***p<0.001 using one-way ANOVA.
