# Supplementary material for: Solid state fermentation process with Aspergillus kawachii enhances the cancer-suppressive potential of silkworm larva in hepatocellular carcinoma cells
Source: BMC Complement Altern Med. 2019 Sep 5;19:241. doi: 10.1186/s12906-019-2649-7 (PMC6727413; doi:10.1186/s12906-019-2649-7)
Supplement: Supplementary file 3 — Table S3. Total polyphenol content from unfermented and fermented silkworm larvae extract. (DOCX 15 kb) [file 12906_2019_2649_MOESM3_ESM.docx]

Supplementary data 3. Total polyphenol content from unfermented and fermented silkworm larvae extract

| Samples | Total polyphenol (mg GAE/g) |
| --- | --- |
| SWE | 4.11 ± 0.14 |
| FSWE | 4.24 ± 0.11 |
| SEE | 3.46 ± 0.09 |
| FSEE | 3.57 ± 0.09 |

The values represent mean±SD of triplicate determinations. GAE, galic acid equivalent; SWE, unfermented silkworm larvae water extract; FSWE, fermented silkworm larvae water extract; SEE, unfermented silkworm larvae ethanol extract; FSEE, fermented silkworm larvae ethanol extract.
